# Supplementary material for: Human mobility on Cancun Island during the Late Postclassic: Intra- and inter-site demographic interactions
Source: PLoS One. 2023 Oct 25;18(10):e0292022. doi: 10.1371/journal.pone.0292022 (PMC10599559; doi:10.1371/journal.pone.0292022)
Supplement: S1 Checklist — (DOCX) [file pone.0292022.s001.docx]

***PLOS ONE* Clinical Studies Checklist**

***PLOS ONE* manuscript number: ___PONE-D-23-08900_________**

| **Complete the following if your study involved human participants or human subjects’ data. These questions should be addressed for prospective and retrospective studies.** | | |
| --- | --- | --- |
| 1. | Did you obtain ethics approval for this study?   - If yes, please upload (file type “Other”) the original approval document you received from your ethics committee. If the original document is in another language, please also provide an English translation.   ___ Uploaded _X__ N/A   - If you did not obtain ethical approval, please explain why this was not required.  \| The human skeletal remains analyzed in the present study are all archaeological specimens recovered from archaeological contexts in Quintana Roo, Mexico. Those persons lived and died between 500 and 1000 years ago. All the human archaeological specimens are protected by the Mexican Federal legislation (Ley Federal sobre Monumentos y Zonas Arqueológicos, Artísticos e Históricos, 1972). All archaeological materials (human or cultural artifacts) are managed and protected by the Instituto Nacional de Antropología e Historia (INAH), which is the Mexican Federal Institution in charge of archaeological materials. As regards the strontium analyses presented in this paper, they were performed under the supervision, permission, and approval of Mexico’s Archaeological Council, which is the INAH’s maximum authority (permission number 401.1S.3-2021/042). In this kind of studies, no ethical approval is required but the approval by the Archaeological Council.  There are NO living human participants involved, NO medical record is reported, NO information is available to identify people who lived 500-1000 years ago, and NO observational study was conducted. Therefore, none of the following points (2 to 5) of this format does apply. \| \| --- \| |  |
| 2. | If your study involved human participants, please report in the Methods section when participants were recruited to the study.  ___ Completed _X__ N/A |  |
| 3. | If you are reporting a study of medical records or archived samples, please report in the Methods section the date range in which human subjects’ data/samples were collected and the date(s) when you conducted this study.  ___ Completed _X__ N/A |  |
| 4. | Please specify in the Methods section whether authors had access to information that could identify individual participants during or after data collection.  ___ Completed _X__ N/A |  |
| 5. | If you are reporting an observational study – i.e. cohort, case-control, and cross-sectional studies – we recommend that the work is reported as per the requirements of the STROBE guidelines, and that you provide a completed STROBE checklist as a Supporting Information file with your submission.  The STROBE checklist was developed to improve the reporting of observational human subjects research, and is available here: <http://strobe-statement.org/fileadmin/Strobe/uploads/checklists/STROBE_checklist_v4_combined_PlosMedicine.docx>.  ___ Completed _X__ N/A |  |
| 6. | Please ensure that the author list and Corresponding Author entered in Editorial Manager match the author list and Corresponding Author in your manuscript file.  __X_ Completed |  |
