# Supplementary material for: Human mobility on Cancun Island during the Late Postclassic: Intra- and inter-site demographic interactions
Source: PLoS One. 2023 Oct 25;18(10):e0292022. doi: 10.1371/journal.pone.0292022 (PMC10599559; doi:10.1371/journal.pone.0292022)
Supplement: Attachment — Submitted filename: Response to reviewers.docx [file pone.0292022.s002.docx]

REPLY TO REVIEWERS

1. Please ensure that your manuscript meets PLOS ONE's style requirements, including those for file naming. The PLOS ONE style templates can be found at
   https://journals.plos.org/plosone/s/file?id=wjVg/PLOSOne_formatting_sample_main_body.pdf and 
   <https://journals.plos.org/plosone/s/file?id=ba62/PLOSOne_formatting_sample_title_authors_affiliations.pdf>

R.**DONE**

2. In your Data Availability statement, you have not specified where the minimal data set underlying the results described in your manuscript can be found. PLOS defines a study's minimal data set as the underlying data used to reach the conclusions drawn in the manuscript and any additional data required to replicate the reported study findings in their entirety. All PLOS journals require that the minimal data set be made fully available. For more information about our data policy, please see http://journals.plos.org/plosone/s/data-availability.

"Upon re-submitting your revised manuscript, please upload your study’s minimal underlying data set as either Supporting Information files or to a stable, public repository and include the relevant URLs, DOIs, or accession numbers within your revised cover letter. For a list of acceptable repositories, please see http://journals.plos.org/plosone/s/data-availability#loc-recommended-repositories. Any potentially identifying patient information must be fully anonymized.

R. TABLES 1 AND 2 REPORT OUR COMPLETE ^87^SR/^86^SR DATASET INCLUDING BOTH HUMAN AND BASELINE VALUES. IT WOULD THEREFORE BE REDUNDANT TO ALSO INCLUDE THE VALUES AS SUPPLEMENTARY INFORMATION FILES. WE HAVE CLARIFIED THIS IN OUR DATA AVAILABILITY STATEMENT.

Important: If there are ethical or legal restrictions to sharing your data publicly, please explain these restrictions in detail. Please see our guidelines for more information on what we consider unacceptable restrictions to publicly sharing data: http://journals.plos.org/plosone/s/data-availability#loc-unacceptable-data-access-restrictions. Note that it is not acceptable for the authors to be the sole named individuals responsible for ensuring data access.

We will update your Data Availability statement to reflect the information you provide in your cover letter.

3. We note that you have stated that you will provide repository information for your data at acceptance. Should your manuscript be accepted for publication, we will hold it until you provide the relevant accession numbers or DOIs necessary to access your data. If you wish to make changes to your Data Availability statement, please describe these changes in your cover letter and we will update your Data Availability statement to reflect the information you provide.

R: SINCE ALL THE INFORMATION ALREADY APPEARS IN THE OPEN-ACCESS MANUSCRIPT, WE CONSIDER IT USELESS TO ADD THE VERY SAME INFORMATION IN A REPOSITORY.

4. Please include your full ethics statement in the ‘Methods’ section of your manuscript file. In your statement, please include the full name of the IRB or ethics committee who approved or waived your study, as well as whether or not you obtained informed written or verbal consent. If consent was waived for your study, please include this information in your statement as well.

R: DONE

5. We note that Figures 1-4 in your submission contain [map/satellite] images which may be copyrighted. All PLOS content is published under the Creative Commons Attribution License (CC BY 4.0), which means that the manuscript, images, and Supporting Information files will be freely available online, and any third party is permitted to access, download, copy, distribute, and use these materials in any way, even commercially, with proper attribution. For these reasons, we cannot publish previously copyrighted maps or satellite images created using proprietary data, such as Google software (Google Maps, Street View, and Earth). For more information, see our copyright guidelines: <http://journals.plos.org/plosone/s/licenses-and-copyright>.

R. FOR FIGURES 1-3, WE HAVE OBTAINED THE PERMISSION FROM THE PERSON WHO MADE THE DRAWINGS. FIGURE 4 HAS BEEN REDRAWN BY THE SENIOR AUTHOR SO NOT TO INCUR IN COPYRIGHT ISSUES. WE ADD FIGURES 7 AND 8 AND WE HAVE ALSO THE PERMISSION FROM THE PERSON WHO MADE THOSE. WE GET THE PERMISSION USING THE CC BY 4.0 LICENSE FOR THESE 5 FIGURES (1 -3 AND 7 AND 8 FIGURES). WE HAVE NOTICE THE FIGURE 1 IS NOT REPRINTED FROM ANY PLACE. IT IS A ORIGINAL FIGURE ELABORATE ON DEMAND OF THE AUTHORS USING A TEMPLATE OF INEGI, THAT IT IS A GOVERNMENTAL INSTITUTION IN MEXICO AND IT IS FREE ACCESS FIGURES 2 AND 3 AND 7 AND 8 ARE DRAWINGS FROM ARCHAEOLOGICAL SITES THAT WERE MADE FOR INSTITUTIONAL PROPOSE AND ARE FREE.

6. Please review your reference list to ensure that it is complete and correct. If you have cited papers that have been retracted, please include the rationale for doing so in the manuscript text, or remove these references and replace them with relevant current references. Any changes to the reference list should be mentioned in the rebuttal letter that accompanies your revised manuscript. If you need to cite a retracted article, indicate the article’s retracted status in the References list and also include a citation and full reference for the retraction notice.

**R: DONE**

Additional Editor Comments:

Thank you for submitting your manuscript to PLOS ONE. 
We now received the reports from both reviewers.
As you will see, they agree about the scientific interest and overall quality of your work.
They do however recommend that some minor issues be addressed (this especially refers to Reviewer 1) before the manuscript be ready for publication.
I encourage you to carefully consider these aspects while preparing the revised version of your submission.
Kind regards
Marco Milella

[Note: HTML markup is below. Please do not edit.]

Reviewers' comments:

Reviewer's Responses to Questions

**Comments to the Author**

1. Is the manuscript technically sound, and do the data support the conclusions?

The manuscript must describe a technically sound piece of scientific research with data that supports the conclusions. Experiments must have been conducted rigorously, with appropriate controls, replication, and sample sizes. The conclusions must be drawn appropriately based on the data presented.

Reviewer #1: Partly

Reviewer #2: Yes

2. Has the statistical analysis been performed appropriately and rigorously?

Reviewer #1: No

Reviewer #2: Yes

3. Have the authors made all data underlying the findings in their manuscript fully available?

The [PLOS Data policy](http://www.plosone.org/static/policies.action#sharing) requires authors to make all data underlying the findings described in their manuscript fully available without restriction, with rare exception (please refer to the Data Availability Statement in the manuscript PDF file). The data should be provided as part of the manuscript or its supporting information, or deposited to a public repository. For example, in addition to summary statistics, the data points behind means, medians and variance measures should be available. If there are restrictions on publicly sharing data—e.g. participant privacy or use of data from a third party—those must be specified.

Reviewer #1: Yes

Reviewer #2: Yes

4. Is the manuscript presented in an intelligible fashion and written in standard English?

PLOS ONE does not copyedit accepted manuscripts, so the language in submitted articles must be clear, correct, and unambiguous. Any typographical or grammatical errors should be corrected at revision, so please note any specific errors here.

Reviewer #1: Yes

Reviewer #2: Yes

5. Review Comments to the Author

Please use the space provided to explain your answers to the questions above. You may also include additional comments for the author, including concerns about dual publication, research ethics, or publication ethics. (Please upload your review as an attachment if it exceeds 20,000 characters)

Reviewer #1: Review of the manuscript entitled:

Human mobility on Cancun Island during the 1 Late Postclassic: intra- and inter-site
demographic interactions - Residential mobility on Prehispanic Cancun Island, Mexico
by A. Cucina et al., submitted to PONE

This is an interesting study that applies Sr isotope signatures of human tooth enamel and bones from two archaeological sites on Cancun island with the aim to assess the presence of non-local people on the East Coast of the Yucatan Peninsula during the Late Postclassic (AD 1200-1540). The very peculiar circumstances that make pinpointing of non-locals to these sites lies in the fact that the local bioavailable baseline, dominated by (sub-) modern marine Sr and by Sr derived from extensive limestone platforms dominating the lowlands of the Yucatan peninsula, is very tightly constrained in its range of 87Sr/86Sr values. The Sr isotope analyses of 50 individuals are complemented with the mortuary distribution, and together with other information on dietary habit, funerary patterns, burial location etc., seven individuals were deemed non-locals that apparently did not receive different treatment and had like been integrated into the local community.

The manuscript is well written and logically built up, and some major issues/drawbacks and challenges with applying Sr isotope provenancing using enamel and bones are brought forward and mentioned in the introductory chapter, more precisely in an own chapter “CONCEPTS OF 87SR/86SR ANALYSIS”.

The abstract is precise and informs the readership in short about the major outcome of the study. The same is valid for the conclusions, which are defined and clearly listed.

Being myself not an archeologist, I cannot judge the validity of the archeological background, but in my opinion the authors give a fair and complete summary of the current stage of knowledge, also in the larger framework of what is known from other sites on Yucatan.

**R: WE ARE GLAD THAT DR FREI FOUND THE MS INTERESTING AND WELL STRUCTURED, AND WE APPRECIATE THE USEFUL COMMENTS THAT TRIGGER AN INTERESTING DISCUSSION AROUND THE ISSUE OF WHO IS SUPPOSED TO BE CONSIDERED “LOCAL”.**

There are two issues I have which the authors will have to comment on, discuss and correct-implement in their revised version.

The first one concerns the definition of the local baseline which the author do not clearly indicate in terms of a statistical reference band. The authors, in the technical chapter, state that “To distinguish non-local individuals, the local 87Sr/86Sr range for Isla Cancun was established using limited faunal baseline data (i.e., archaeological opossum [Didelphis sp.] 87Sr/86Sr = 0.7092) and supplemented by previously published values from Mesoamerica (e.g., [31] [32] [45]).”

What exactly is this baseline in 87Sr/86Sr space? It looks to me that the authors chose to make a cut-off at around 87Sr/86Sr ~ 0.70901 (they call it plateau in their Figure 5), which they support by the faunal value they measured on the one opossum). This value is very close to the modern marine signature, and it is doubtful that this cut-off pays respect to the likely influence of very nearby Tertiary limestone dominated bioavailable Sr (for example on drinking water), also in the past. I would strongly suggest the authors broaden their baseline range and incorporate very local signatures of Sr published not so long ago by L.M. Hernandez-Terrones et al. (2021), Continental Shelf Research 212, 104293 (if I am correct, the authors are not citing this work in the submitted manuscript…). This paper summarizes a larger number of environmental 87Sr/86Sr values from the immediate Cancun area, also form the coast near mainland, and not only form the lagoon. I have calculated the average 87Sr/86Sr value of 0.70884 +/- 0.00072 (2s) from the data in this paper and strongly advocate using this range as a conservative and representative range of bioavailable e Sr instead. If implemented, and also indicated in Figure 5, then it becomes clear that the number of non-locals decrease, and locals then are not those that lived on the lagoon only but leaves ample space to also include individuals that lived along the mainland coast just around Cancun where the Tertiary carbonates are prevalent. I could imagine a scenario where individuals could easily move from the mainland coast to the logon and back and that the term “local” would still be applicable under such circumstances.

R: WE DO APPRECIATE DR. FREI’S COMMENTS, WHICH GIVE US A HINT TO IMPROVE THE MANUSCRIPT AND CONTRIBUTE TO SOME EXTENT TO AN INTERESTING ACADEMIC DISCUSSION ABOUT WHO IS LOCAL AND WHO IS NOT BASED ON CLOSE SR (AND OXYGEN) VALUES. THERE IS STILL SO MUCH TO DISCOVER ABOUT ISOTOPIC VALUES IN EACH REGION, AND IT IS TRUE THAT MOBILITY WAS INTENSE ALSO IN PREHISPANIC TIMES. WE ARE AWARE THAT BASELINE DATA TO ESTABLISH “LOCALS” VERSUS “NON-LOCALS” IS A COMPLEX ISSUE. WE ARE ALSO VERY GRATEFUL TO DR. FREI TO REFER TO US THE PAPER BY HERNÁNDEZ-TERRONES ET AL. (2021), WHICH WE CONSIDER USEFUL TO SUPPORT OUR HYPOTHESIS.

WE CROSS-CHECKED THE WATER SAMPLES ANALYZED BY HERNÁNDEZ-TERRONES ET AL. (2021) WITH THE GEOGRAPHICAL LOCATION THEY PROVIDED, BASED ON GOOGLE EARTH. ALL THE SAMPLES FROM CANCUN/PUERTO MORELOS WERE COLLECTED IN AN AREA RANGING BETWEEN 20.84295 TO 21.09336 (LATITUDE), AND 86.81569 AND 86.91689 (LONGITUDE), WHICH ENCOMPASSES INLAND TERRITORIES THAT ARE BETWEEN 16 AND 22 MILES DISTANT FROM THE COASTAL SHORES WHERE CANCUN ISLAND IS LOCATED. THOSE PLACES SHOW A WIDE RANGE OF SR RATIOS; HOWEVER, NO WATER SOURCE HAS BEEN SAMPLED CLOSE TO THE COAST, SO IT IS DIFFICULT TO KNOW THE EXTENT OF VARIABILITY OF THE POTENTIAL WATER SOURCES ALONG THE COAST. IN OUR PERSPECTIVE, IT IS DIFFICULT TO THINK THAT PEOPLE LIVING ON THE COAST WERE RELYING ON WATER SOURCES LOCATED SO FAR INLAND. INSTEAD, THEY MUST HAVE RELIED ON OTHER NOT-YET-SAMPLED WATER SOURCES, AND AT CANCUN ISLAND THERE ARE MANY UNDERGROUND WATER-STORAGE DEPOSITS (CHULTUNES).

GIVEN THE LACK OF WATER/PLANT/GEOLOGICAL SOURCES FOR THE EAST COAST, THE 0.7091 OVERALL VALUES THAT WE CONSIDER AS REPRESENTATIVE OF THE LOCAL PEOPLE ARE THOSE THAT HAVE BEEN CONSISTENTLY REPORTED IN THE MAJORITY OF THE HUMAN CASES (AND ANIMAL SAMPLES) BOTH AT ISLA CANCUN, AT EL MECO, LOCATED JUST NORTH OF CANCUN, AND TULUM. IN FACT, THE AVERAGE VALUE FOR THOSE SUPPOSEDLY LOCAL PEOPLE AT CANCUN ISLAND AND EL MECO (SEE ORTEGA ET AL. 2019) IS 0.70916, WHICH IS WELL ABOVE FROM THE SUGGESTED LOWER LIMIT OF 0.7088.

WE HAVE REPLACED ONE FIGURE (AS REQUESTED BY REVIEWER N. 2), SHOWING THE RANGE OF DISTRIBUTION PER SITE. IN THIS PERSPECTIVE, IT IS INTERESTING TO NOTE THAT, WITH ONLY ONE EXCEPTION OF AN INDIVIDUAL WHOSE SR VALUE IS 0.7088 (THAT HAS BEEN CONSIDERED AS NON-LOCAL ALSO BASED ON HIS OXYGEN VALUE), NO OTHER PEOPLE PRESENTED SR RATIOS BELOW 0.7090. IF THE LOWER LOCAL VALUE WERE, INDEED, 0.7088 (ASSUMING THE LOGICAL MOVEMENT TO AND FROM THE COAST), THEN WE SHOULD EXPECT A MUCH LARGER NUMBER OF PEOPLE WITH SR RATIOS BETWEEN 0.7088 AND 0.7091. INSTEAD, THIS DOES NOT HAPPEN; ONLY ONE INDIVIDUAL - THE ABOVEMENTIONED 0.7088 FOLK - AND TWO INDIVIDUALS WHOSE SR RATIO OF 0.70903 IS SUGGESTIVE AS POTENTIALLY NON-LOCAL, FALL BETWEEN THE 0.7088 AND 0.7091 RANGE, OUT OF 44 (OUT OF 50) MORE PEOPLE FROM THE COAST WITH VALUES ABOVE THE 0.7091 THRESHOLDS (PLUS 13 FROM EL MECO, AND 3 FROM TULUIM – ORTEGA MUÑOZ ET AL. 2019). THEREFORE, WE TRULY THINK THAT THE LOCAL SR RATIO “ALONG” THE COAST (I.E., PEOPLE WHO WERE BORN ON THE COAST, AND NOT 16 TO 22 MILES INLAND), IS AROUND 0.7091. RESTING ON DR. FREI’S COMMENTS, THE QUESTION THAT WE ADDRESS IS: WHICH PEOPLE SHOULD BE CONSIDERED AS “LOCALS” IN CONTRAST WITH “NON-LOCALS”? WHICH IS THE MINIMUM DISTANCE FROM THE COASTAL SHORE OF THE EAST COAST FOR A PERSON TO BE CONSIDERED AS A LOCAL? OUR POSITION IS THAT WE DO NOT THINK THAT PEOPLE WHO POTENTIALLY GREW UP SOME CA. 20 MILES INLAND SHOULD BE CONSIDERED AS “LOCALS” TO THE EAST COAST’ SHORES. AND, BASED ON THE DATASET AVAILABLE, THERE ARE SO FEW PEOPLE WITH SR RATIOS BELOW 0.7090 TO CONSIDER THE 0.7088 VALUE AS THE MINIMUM FOR THE LOCAL RANGE.

ON THE OTHER HAND, WE THINK, THOUGH WE TREAT THEM WITH CAUTION (AND WE MAKE IT CLEAR IN THE TEXT) THAT THOSE TWO PEOPLE WITH VALUES CLOSE TO THE 0.7090, WHOM WE CONSIDER AS POTENTIALLY NON-LOCALS OF THE EAST COAST’ SHORES, ORIGINATED FROM PLACES NEARBY CANCUN ISLAND.

The second issue I would like to point out and where I would propose the authors come in a bit in more detail in their discussion of the data, is the fact that in the instances where tooth enamel and bone form the same individual were analyzed, it becomes apparent that the bones nearly consistently exhibit a marine Sr isotope signal which in some cases differs strongly form the respective tooth enamel with lower 87Sr/86Sr values. As hinted by the authors vaguely, this to me is a clear sign of diagenetic contamination of the original bone signatures, a fact well known by respective research community. I sternly encourage the authors to discuss this outcome in more detail, also with respect to using such data for the provenance aspects. Also here, it would be very informative if the authors added a realistic baseline range in their Figure 6.

R: DR. FREI IS PERFECTLY RIGHT WHEN ADDRESSING THE DIAGENETIC ISSUE IN HUMAN BONE. AS SUGGESTED, WE HAVE ADJUSTED OUR INTERPRETATION TO IT. UNFORTUNATELY, WE DO NOT HAVE MUCH MORE INFORMATION ON BONE SR RATIOS TO PROVIDE A MORE DETAILED DISCUSSION SINCE THE METHODOLOGICAL ISSUE OF RECOVERING AND CONFIRMING NON-DIAGENETIC ^87^SR/^86^SR FROM BONE APATITE REMAINS UNDER DEBATE (E.G., SEE WATHEN, C.A., ISAKSSON, S. & LIDÉN, K. ON THE ROAD AGAIN—A REVIEW OF PRETREATMENT METHODS FOR THE DECONTAMINATION OF SKELETAL MATERIALS FOR STRONTIUM ISOTOPIC AND CONCENTRATION ANALYSIS. *ARCHAEOL ANTHROPOL SCI* **14**, 45 (2022). HTTPS://DOI.ORG/10.1007/S12520-022-01517-2

All in all, I enjoyed reading through this manuscript and I recommend publication with moderate revisions along the critical points mentioned above.

R: AS SAID, WE REALLY APPRECIATE DR. FREI’S USEFUL COMMENTS, AND WE ARE GRATEFUL FOR THAT.

11.4.2023 Robert Frei

Reviewer #2: This is a well researched and written paper, and it was such a joy to read your work! Overall, I think that this contribution is vital to bio(archaeological) research - I have a few suggestions/considerations that I think would be helpful to discuss.

A) For Table 3, you present the descriptive statistics of the isotope ratios from San Miguelito and El Rey - Making a box plot graph based on this tables data will also show the slight differences between your groups and adds to your argument about differences/similarities. I would think this would add additional context to the 2 towns/1 town discussion you had as well.

R: FIGURE 5 HAS BEEN REDRAWN COMPLETELY, AND NOW IT INCLUDES ALSO A BOX-PLOT (FIG. 5B). A SENTENCE HAS BEEN ADDED TO THE DISCUSSION TO REFER TO THE TWO SITES AS BEING ONE OR TWO SEPARATE POLITICAL ENTITIES.

B) Would it be possible to include a map with the structures, and the local/non-local and see if there is a mortuary pattern based on the structure? For example, Burial 26 (5 years, potential non local) and Burial 2 (adult, local) come from structure 1 - so I wonder if you could make a bigger deal about the implications of this data, based on spatial analysis to illuminate other potential social structures?

R: WE INCLUDE FIGURES 7 AND 8 THAT INCLUDE THE LOCATION OF BURIALS AND THEIR PROVENANCE. DISCUSSION HAS BEEN IMPROVED BASED ON IT.
