# Supplementary material for: Human mobility on Cancun Island during the Late Postclassic: Intra- and inter-site demographic interactions
Source: PLoS One. 2023 Oct 25;18(10):e0292022. doi: 10.1371/journal.pone.0292022 (PMC10599559; doi:10.1371/journal.pone.0292022)
Supplement: Attachment — Submitted filename: Reponse to reviewers.docx [file pone.0292022.s003.docx]

REBUTAL LETTER

Figure 5A: It is not quite clear to me what you are actually showing in it from the relative legend. What are the 2 axes representing? Could you color somehow differently the different sites? Also, it may be beneficial to explicitly indicate in the plot the outliers burial id (rather than isotopic values: these you already have in the tables).

R. We change the Figure 5A to bar graph, because, the authors are agreeing that is an easier way to show and comprehend the strontium stable isotopic ratios, with the axe-y with the values of ^87^Sr/^86^Sr. We highlight in gray color the non-locals, and We separate San Miguelito from El Rey

Figure 5B (I am confused by the numbers indicating the outliers: maybe this is a typo and by mistake you indicated the row numbers in SPSS or other variable?).

R. Yes it was a mistake We had done again the Figure, with the indicating burial ID.

Figure 6: this is an unusual way to graphically report isotopic values. I suggest you to either plot bone vs enamel values (in this case, residuals from perfect correlation indicate isotopic differences, and their relative direction), or in any case present these data as points (x axis would be individual number, y axis strontium values, each point a sample and 2 different markers for bone and enamel).

R. Yes, Figure 6 has been redone following the suggestion

In your graphs (especially figures 5B and 6) please use the standard notation for strontium isotopic ratios **(87Sr/86Sr)**.

R. DONE

**Reviewers' comments:**

Reviewer's Responses to Questions

**Comments to the Author**

Reviewer #1: Dear authors,

you have satisfactorily addressed my two main questions regarding the definition of the baseline and the diagenetic issue, respectively. Please correctly state the baseline range!! you deem appropriate on line 393 after the "+".

R. Done. It was I mistake of our part.

The added discussion of the Hernandez data and the problem of diagenesis is now at least there, although I am personally still not convinced entirely about the definition of the baseline range. surely some local baseline data are lacking, and maybe this should be added in the text as well!

R. We are completely in agreement with you. More research in this perspective about new data to reenforce the baseline in coastal and inland areas of the peninsula of Yucatan, Mexico, is needed. Mainly to evaluate the diagenesis process in bones and teeth in human and faunal of the region.

Please also be aware that the Waten paper discusses how bones that that were treated for preservation in respective museums can be cleaned from glue to reveal useful bioavailable Sr signatures, these authors do not discuss on how to recover primary Sr isotope signatures from in situ diagenetic contamination which your bones clearly show. Maybe is can be taken positively, to support your point, that the local Sr signature is actually preserved in the diagenetically alter bones instead?

R. The samples process to Sr analysis were not treated with any chemical products (glue, mowital, or others). The cleaning process was with distilled water to remove the dirt or soil matrix. We discard diagenetic contamination both from the place that these individuals were buried because the isotopic signature is diverse, but with small range. For example, Price and colleagues (2020: 592) show that the values of ^87^Sr/^86^Sr of bones samples of sailors of the voyages of Columbus to America, buried in the Santo Domingo Island, are generally homogenous (mean: 07092+- 0.00002), and they consider that these samples are contaminated by local strontium isotopes. Our samples have a mean of 0.70918+- 0.00003, (min=0.709119; max= 0.709214) and from fauna is 0.70920. Then, the diagenetic contamination could not be discarded. We have pointed out this aspect about contamination in the manuscript and its limitation t implies, at least for the bony remains.
